# Targeting Phosphatases and Kinases: How to Checkmate Cancer

**DOI:** 10.3389/fcell.2021.690306

**Published:** 2021-10-28

**Authors:** Alice Turdo, Caterina D’Accardo, Antonino Glaviano, Gaetana Porcelli, Cristina Colarossi, Lorenzo Colarossi, Marzia Mare, Naida Faldetta, Chiara Modica, Giuseppe Pistone, Maria Rita Bongiorno, Matilde Todaro, Giorgio Stassi

**Affiliations:** ^1^Department of Health Promotion, Mother and Child Care, Internal Medicine and Medical Specialties (PROMISE), University of Palermo, Palermo, Italy; ^2^Department of Surgical, Oncological and Stomatological Sciences (DICHIRONS), University of Palermo, Palermo, Italy; ^3^Department of Experimental Oncology, Mediterranean Institute of Oncology (IOM), Catania, Italy; ^4^Villa Sofia-Cervello Hospital, Palermo, Italy; ^5^Azienda Ospedaliera Universitaria Policlinico (AOUP), Palermo, Italy

**Keywords:** phosphatase, kinase, cancer stem cell, phosphatase and kinase inhibitors, targeted therapies

## Abstract

Metastatic disease represents the major cause of death in oncologic patients worldwide. Accumulating evidence have highlighted the relevance of a small population of cancer cells, named cancer stem cells (CSCs), in the resistance to therapies, as well as cancer recurrence and metastasis. Standard anti-cancer treatments are not always conclusively curative, posing an urgent need to discover new targets for an effective therapy. Kinases and phosphatases are implicated in many cellular processes, such as proliferation, differentiation and oncogenic transformation. These proteins are crucial regulators of intracellular signaling pathways mediating multiple cellular activities. Therefore, alterations in kinases and phosphatases functionality is a hallmark of cancer. Notwithstanding the role of kinases and phosphatases in cancer has been widely investigated, their aberrant activation in the compartment of CSCs is nowadays being explored as new potential Achille’s heel to strike. Here, we provide a comprehensive overview of the major protein kinases and phosphatases pathways by which CSCs can evade normal physiological constraints on survival, growth, and invasion. Moreover, we discuss the potential of inhibitors of these proteins in counteracting CSCs expansion during cancer development and progression.

## Introduction

The main role of kinases and phosphatases is to regulate post-translational modifications of proteins, which are essential to govern cellular signaling networks (Sacco et al., 2012). Mutations in kinases (Irby et al., 1999; Zhang et al., 2009) or phosphatases (Stebbing et al., 2014; Zhao et al., 2015) that lead to either a loss-of-function or gain-of-function are likely to cause cancer. Moreover, oncogenes can influence the balance between kinases and phosphatases activity, causing cell malignant transformation (Qi et al., 2018). The uncontrolled activation of kinases and the suppression of phosphatases has been frequently observed in cancer with consequent induction of cell proliferation, migration and survival to anti-cancer therapies. Failure in the balance between kinases and phosphatases activity has been shown in several types of solid cancer, such as colorectal, gastric, liver and breast cancer (BC) (Martellucci et al., 2020). Thus, it is plausible that a better understanding of how kinases and phosphatase enzymes function and how they are regulated can aid the development of new anticancer agents.

Cancer recurrence and relapse are attributed to a small subpopulation of cancer cells, named cancer stem cells (CSCs) (Turdo et al., 2020). CSCs are also able to self-renewal and multilineage differentiation, as well as an ability to initiate and support tumorigenesis and metastasis formation (Veschi et al., 2020b).

This review focuses on dysregulation of kinase and phosphatase activity, since they represent the major players that sustain CSCs persistence in a variety of cancers. Moreover, herein we will discuss novel therapeutic compounds that inhibits kinase and phosphatase proteins involved in carcinogenesis.

## The Role of Kinases and Phosphatases in Cancer

Kinases and phosphatases carry out essential roles in a plethora of biological functions and regulatory network of cells (Ostman et al., 2006; Ventura and Nebreda, 2006; Malumbres and Barbacid, 2009; Bononi et al., 2011; Otto and Sicinski, 2017). Kinases catalyze the transfer of phosphate groups, released by ATP, to molecules while phosphatases remove phosphate groups from their substrate proteins.

Kinase phosphorylation can modify the function of a protein by increasing or decreasing its activity, enhancing its stabilization, marking it for destruction, localizing it within a specific cellular compartment, and initiating or disrupting its interaction with other proteins. Protein kinases often act on multiple substrates and different proteins can serve as substrates for more than one specific kinase. Kinases mediate most of the signal transduction of the cell, and consequently, control many cellular processes, including transcription, proliferation, apoptosis, metabolism, interplay with the immune systems, migration, cytoskeletal rearrangement and differentiation (Rubin et al., 2000; Lander et al., 2001; Venter et al., 2001). The largest group of kinases is composed by protein kinases, which phosphorylate proteins at serine/threonine, tyrosine or all three residues (dual-specificity kinases) (Krupa et al., 2004).

The family of protein tyrosine kinases consists of the receptor tyrosine kinases (RTKs) proteins and the non-receptor tyrosine kinases (nRTKs). Besides regulating several cellular processes in normal cells, RTKs are implicated in the development and progression of cancer. Indeed, mutations in RTKs lead to the constitutive activation of the receptor and uncontrolled activation of multiple signal transduction pathways (Schmidt-Arras and Bohmer, 2020). Almost 20 different RTKs classes have been described, which include EGFR, insulin R, PDGFR, VEGFR, FGFR, HGFR and RET (Blume-Jensen and Hunter, 2001). Differently from RTKs, the nRTKs are cytosolic enzymes, in some cases anchored to the cell membrane. Janus kinase (JAK) and Src families are the most important nRTK families involved in cancer. The JAK proteins transduce signals that are mediated by cytokines in the JAK-STAT pathway while Src, when activated, is known to phosphorylate PI3K, RAS and STAT to promote proliferation, survival and invasion of cancer cells (Kisseleva et al., 2002; Ishizawar and Parsons, 2004).

Among the protein serine/threonine kinases, PKA, MAPKs, RAF, PKB (also known as Akt), GSK-3, mTOR and cyclin-dependent kinases (CDKs) are among the most frequently occurring drivers of human cancer.

The major function of PKA in the cell include regulation of carbohydrate and lipid metabolism (Turnham and Scott, 2016). The MAPK is a complex series of signal transduction pathways connecting extracellular signals to intracellular responses, whose function is the regulation of important processes such as cell proliferation, differentiation, and death (Raman et al., 2007). During the last decades, the insight of each main MAPK signaling modules and their role in tumorigenesis has grown remarkably (Wagner and Nebreda, 2009). The four main MAPK signaling modules are (i) ERK1/2, (ii) JNK, (iii) p38, and (iv) ERK5 pathways, which are induced by specific extracellular signaling. RAF kinase is activated by growth factors, forms part of the RTKs/RAS/RAF/MEK/ERK pathway and its leading function is to stimulate cell division and growth (Chang and Karin, 2001). Moreover, the majority of solid tumors are explicitly characterized by their overall mutations along all the RTKs/RAS/RAF/MEK genes of the signaling pathway (Stern, 2018). Interestingly, MEK kinase is a component of the MAPK pathway which acts on both serine/threonine and tyrosine kinase residues.

GSK-3 is considered to be at the crossroads of various cancer pathways and a major component of the RTKs/RAS/PI3K/PTEN/Akt/GSK-3/mTORC1 axis. Upon Akt-mediated phosphorylation, GSK-3 is inactivated and targeted for proteasome degradation (Cross et al., 1995). As Akt is often active in human cancer, GSK-3 is consequently often inactivated. GSK-3 can also regulate NF-κB and WNT/β-catenin pathway activity (Gotschel et al., 2008).

Cyclin-dependent kinases are intracellular serine/threonine protein kinases whose central activity is the regulation of the cell cycle. Therefore, they are responsible for the progress of the cell through its various checkpoints (Harper and Adams, 2001). Dysregulation of CDKs, such as CDK1, CDK2, CDK3, CDK4 and CDK6, is a well-known hallmark of cancer. Several studies have focused on trying to establish a strategy to inhibit specific CDKs proteins involved in cell cycle progression, leading to uncontrolled cell proliferation in many solid cancer types (Asghar et al., 2015).

Lipid kinases are a smaller group of kinases that add phosphate groups to lipids causing a change in the reactivity and localization of the lipid with a consequent modulation of signal transmission (Whitman et al., 1988). Phosphoinositide 3 kinase (PI3K) is the main player of the PI3K/Akt pathway, which is the most common activated signaling in human’s cancer (Lawrence et al., 2014). This signaling network is activated downstream of RTKs and regulates cell survival, growth, transcription and protein synthesis (Fruman et al., 2017). Phosphatases are known to exert dephosphorylation on RTKs, whose activity can be modified in both a positive and negative manner (Volinsky and Kholodenko, 2013). Consequently, phosphatase dysregulation can hamper RTK regulation, emphasizing their critical implication in the onset and progression of cancer (Yao and Stagljar, 2017).

A total of 211 phosphatase domains have been characterized and then assigned to six different families, defined by catalytic domain sequence similarity (Sacco et al., 2012). The major categories include the phosphoprotein phosphatase (PPP) and the protein phosphatase Mg^2+^- or Mn^2+^- dependent (PPM) families that dephosphorylate phosphoserine and phosphothreonine residues, the haloacid dehalogenase (HADS), and the most dominant group of Cys-dependent protein tyrosine phosphatase (PTP) family that dephosphorylate phosphotyrosine amino acids. Notably, a subfamily of the PTPs, the dual-specificity phosphatises (DUSPs), dephosphorylate all three phosphoamino acids (Alonso et al., 2016).

The PPM family component PPM1D, also termed WIP1, is nowadays considered as an oncoprotein because of its negative regulation of target anti-cancer proteins such as p53, ATM, H2AX and p16. Amplifications and mutations of PPM1D has been frequently observed in cancer, and linked to the progression of the disease and therapy resistance phenomena (Deng et al., 2020b).

The phosphatase PP2A, belonging to the PPP family, is the most expressed serine/threonine phosphatase in mammalian cells and plays a fundamental role in the control of normal kinases activity (Westermarck and Hahn, 2008). PP2A has been primarily described as a tumor suppressor involved in diverse signaling networks regulating cancer progression (Mumby, 2007). The main PP2A function is to inhibit the RAF-MEK-ERK pathway (by reducing activity of both ERK and RAF), and to dephosphorylate and inhibit Akt, c-Myc, and RalA (Zhang and Claret, 2012).

Among the classical PTPs, the receptor-type T PTP (PTPRT) and the receptor-type D PTP (PTPRD) have been reported to be involved in the negative regulation of the JAK/STAT pathway and in particular of STAT3 (Veeriah et al., 2009). Both PTPRT and PTPRD have been described as tumor suppressors in a variety of cancers (Wang et al., 2004; Funato et al., 2011). Moreover, it has been reported that PTPRH and PTPRB directly exert dephosphorylation on EGFR, and thence, suppress its downstream signaling in PI3K/Akt/mTOR and MEK/MAPK pathways (Yao et al., 2017).

The classical PTPs non-receptor type comprise the PTP1B, whose activity has been associated with poor prognosis in breast, gastric, colorectal, hepatocellular and lung cancer by regulating variable tumor-specific mechanisms (Bollu et al., 2017). Also, it has been shown that SHP2 (also known as PTPN11), is a main functional regulator of RTK. Indeed, somatic *PTPN11* mutations has been linked to human malignancies, including juvenile leukemia and juvenile myelomonocytic leukemia (Grossmann et al., 2010). Finally, it has been suggested that protein *PTPN13* is a tumor-suppressor gene, for instance in non-small cell lung cancer, likely due to the control of phosphorylation of both RTK type receptors EGFR and HER2 (Scrima et al., 2012).

It has been shown that numerous DUSP are critical regulators of the MAPK family, which includes ERK and JNK (Liu et al., 1995; Muda et al., 1996; Nunes-Xavier et al., 2013). Besides, PTEN, which is a crucial member of the DUSP family (Myers et al., 1997), negatively regulates intracellular levels of PIP3 and functions as a tumor suppressor by exerting a negative regulation on the PI3K/Akt signaling pathway (Lee et al., 2018). Loss of PTEN, which results in hyperactivation of PI3K pathway, and thus an increase in cell proliferation, has been identified as a decisive genetic event triggering the onset of a wide variety of neoplasm. PTEN activity includes the control of apoptosis, migration, metabolism and anti-cancer therapy response of cancer cells (Lee et al., 2018).

PTEN has been frequently found to be downregulated by PRL-3 in colorectal cancer (CRC). Overexpression of phosphatase PRL-3 is associated with activation of the PI3K/Akt pathway, which can promote epithelial to mesenchymal (EMT) and tumor progression (Wang et al., 2007).

The CDK-associated protein phosphatase (KAP) is overexpressed in cancer cells. It participates to the G1/S transition of the cell cycle and forms a complex with CDK2. Indeed, KAP promotes growth of cancer cells and determines resistance to anti-tumor necrosis factor-α-induced apoptosis by preventing the activation of caspase-3 (Lai et al., 2012). Additionally, cells overexpressing KAP show a higher ability of cell invasion and tumorigenicity (Stebbing et al., 2014).

A class of DUSP proteins involved in the regulation of MAPK pathway, thus also referred to as MAP kinase phosphatases (MPK), dephosphorylate ERK, JNK and p38 at tyrosine and serine/threonine residues. In physiological conditions, DUSP expression levels are positively regulated at transcriptional level by ERK, generating a negative feedback loop to restrain RAS signaling. DUSPs are classified, in more than ten members, according to the specificity of the substrate and cell localization (Caunt and Keyse, 2013). DUSPs are generally defined as tumor suppressor even though several evidence reported a pro-tumorigenic role (Furukawa et al., 2003). For instance, DUSP1 and DUSP6 inhibition suppresses tumor growth in leukemia and BC. Indeed, in these cases DUSP activity seems to favor cancer cell adaptation to high proliferative stimuli (Kaltenmeier et al., 2017; Kesarwani et al., 2017).

Cyclin-dependent kinases dysregulation, which is often determined by altered phosphatases, is undoubtedly a notorious hallmark of cancer. As a result, it is important to understand the abnormal role of specific proteins, including phosphatases, which can facilitate cell cycle progression, leading to uncontrolled cell proliferation and malignancy (Asghar et al., 2015). Cell division cycle 25 (CDC25) families are DUSPs and determine the activation of CDKs, which in turn regulate cell-cycle progression. Three isoforms have been characterized CDC25A, CDC25B and CDC25C, which showed a correlation with poor survival and multi-drug resistance (Galaktionov et al., 1995; Karagoz et al., 2010; Albert et al., 2012).

Given the indispensable role of kinase and phosphatase enzymes in cancer onset and progression (an extensive review literature can be found in the following references Ostman et al., 2006; Ventura and Nebreda, 2006; Malumbres and Barbacid, 2009; Bononi et al., 2011; Otto and Sicinski, 2017), several research studies, as reported below, are nowadays aimed at uncovering the contribution of these enzymes in cancer stemness.

## Kinases and Phosphatases are Crucial Players in Cancer Stem Cells

**FIGURE 1:**
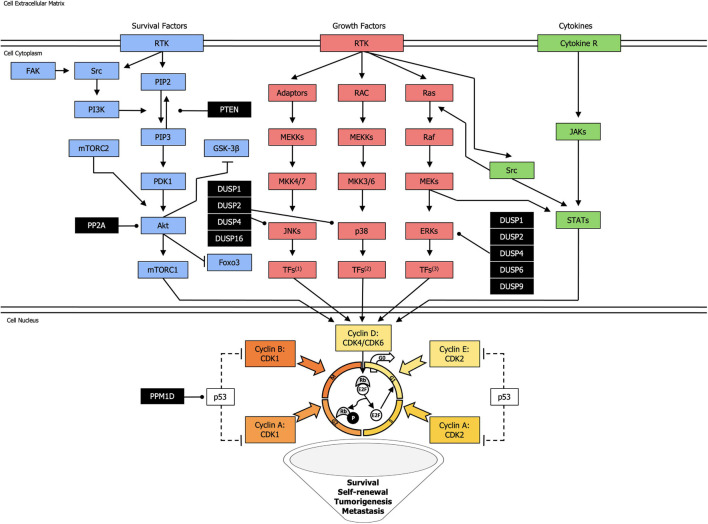
Landscape of the most common kinases and phosphatases network in cancer stem cells. Kinase activating phosphorylation is shown with arrowed lines, inhibitory phosphorylation or non-phosphorylation is indicated with blocked lines, and dephosphorylation, exerted by phosphatases (black squares), is displayed with round-ended lines. Dotted lines represent indirect effect. TFs^1^: Transcription Factors (Jun, ATF2, RNPK, p53, NFAT4, Shc); TFs^2^: Transcription Factors (CHOP, ATF2, MNK, MSK, MEF2, Elk-1); TFs^3^: Transcription Factors (Elk-1, Ets-2, RSK, MNK, MSK, cPLA2).

Cancer is a heterogeneous disease at phenotypic and genetic level. Many studies have investigated the role of CSCs in cancer progression and resistance to therapy. Kinases and phosphatases play important roles in maintaining CSC phenotypes, including self-renewal capacity, invasiveness, and tumorigenicity (Figure 1).

The PI3K/Akt pathway is among the best investigated in human biology, and its pathological activation is considered as a “driver” in numerous cancers. Growing evidence suggests an important role for PI3K signaling in the regulation of stemness, and the underlying mechanisms are still under investigation (Todaro et al., 2014; Di Franco et al., 2016; Mangiapane et al., 2021). Previously Dubrovska et al. (2009) showed that PTEN/PI3K/Akt pathway is critical for prostate CSCs maintenance. Prostate cancer cells treated with the PI3K inhibitors showed a reduced capacity to form spheres. Conversely, genetic silencing of *PTEN*, caused a significant increase of cancer progenitors and stem-like cells in prostate cancer (Dubrovska et al., 2009) and glioblastoma (Duan et al., 2015). Other activities in CSCs are mediated by the activation of PI3K/Akt/mTOR pathway. In particular, mTOR pathway has a central role in the maintenance of CSCs. The PI3K and mTOR pathway sustained the expansion of side population cells, expressing a CD44^+^/CD24^–^ phenotype, in MCF7 BC cell lines, and fostered tumorigenic potential *in vivo* (Li et al., 2018). More recently, it has been shown that the activation of PI3K/Akt pathway characterized breast CSCs (BCSCs) expressing high levels of multidrug resistance (MDR) (Hu et al., 2015). The PI3K/PTEN/Akt/mTOR signaling regulated CSC activity in gefitinib-resistant A549 cells, which contained a high proportion of CXCR4^+^ cells endowed with an enhanced potential of self-renewal activity *in vitro* and tumor growth *in vivo* (Jung et al., 2013).

Chang et al. (2015) found that prostate cancer radioresistance is sustained by the activation of a stemness and EMT phenotype dictated by the activation of the PI3K/Akt/mTOR signaling pathway. PI3K/mTOR also positively regulated aldehyde dehydrogenase 1, member A1 (ALDH1A1) expression and ALDH activity, through SOX9 transcriptional activation, in head and neck squamous cancer cell (Keysar et al., 2017). PI3K/Akt pathway likely contributes to survival and resistance of brain CSCs. Specifically, among the PI3K isoforms, the p110α was decisive to sustain sphere formation and clonogenic capability of medulloblastoma cells. Notably, the PI3Kα catalytic isoform acted in synergism with MAPK-interacting kinase (MNK) to enhance medulloblastoma stem-like properties (Eckerdt et al., 2019).

Also MAPK signaling is involved stem cell biology. Blaj et al. (2017) showed that MAPK pathway is significantly related to intratumoral heterogeneity of CRC. The oncogenic effect of MAPK activity appeared consistently restricted to tumor cells placed at the leading edge, whereas more differentiated tumor cell placed at the central core of the tumor had lower MAPK activity. Moreover CRC cells, displaying high MAPK activity, had a distinct phenotype characterized by decreased epithelial markers expression, such as E-cadherin, and increased expression of the LAMC2 mesenchymal marker, which is a target of ZEB1 (Blaj et al., 2017). Recently it has been demonstrated in CRC that MAPK and FAK signaling pathways are able to maintain the ALDH^+^ cell population, which has been described in various cancer, such as colon, breast, lung, head and neck squamous cancer, to possess tumor-initiating capabilities (Tomita et al., 2016).

JAK-STAT3 pathway regulated CSCs properties of thyroid anaplastic carcinoma. Anaplastic thyroid carcinoma is one of the most aggressive carcinoma refractory to current therapies. CSCs, responsible for his high malignancy, have been previously identified and characterized (Todaro et al., 2010). *In vitro* experiments have showed that JAK1 inhibitor suppressed specifically the stem cell compartment of THJ16T cells, suggesting a role of JAK/STAT pathway in thyroid CSCs growth (Shiraiwa et al., 2019). Zhou et al. (2007) demonstrated that the JAK/STAT3 pathway, in synergism with mTOR, was fundamental for breast cancer stem-like cell survival *in vitro* and in a preclinical model of nude mice. The screening of a large scale library of shRNAs revealed that the JAK2/STAT3 signaling was selectively activated in CD44^+^/CD24^–^ BCSCs as compared with the differentiated compartment (Marotta et al., 2011). Similarly, IL-6 secreted by CRC-derived mesenchymal stem cells promoted the onset of a stemness phenotype in CRC cells through the activation of JAK2/STAT3 pathway (Zhang et al., 2018). Tumor micro environmental cues are indeed the major upstream activators of the JAK/STAT pathway. Yang et al. (2019) demonstrated that IL-10 derived by tumor associated macrophages dictates the stem cell fate of non-small cell lung cancer (NSCLC) cells *via* the activation of JAK1/STAT1 signaling. Under the hypoxic tumor microenvironment, hypoxia-inducible factor 1 α (HIF-1α) activated the JAK1-2/STAT3 pathway in glioma stem-like cells causing an enhancement in self-renewal and a delay in *in vivo* tumor growth (Almiron Bonnin et al., 2018).

Recently, by using an unbiased proteomic profiling combined to *in vivo* transplantation studies, Coles et al. (2020) showed that PKA is a key kinase responsible for initiation and progression of small cell lung cancer (SCLC). Activation of PKA activity in SCLC cells increased significantly the stem cell frequency, the expression of stem cell markers, such as NCAM1, DLL3, MYCL and CD24 and tumor growth of xenografts. Beyond the role of PKA in SCLC pathogenesis, these data provided essential insights regarding PKA signaling networks, comprising the G-protein α subunit and the serine/threonine protein phosphatase PP2A as positive and negative regulators respectively (Coles et al., 2020).

Phosphatases have been also linked to stem cell biology. SHP2, currently under investigation for therapeutic proposal, has a specific role in regulating CSCs biology. The function of SHP2 within CSCs has been characterized in several tumors, such as leukemia, BC, glioma and liver carcinoma. Aceto et al. (2012) showed that SHP2 was able to influence the self-renewal of tumor cells. In fact, *SHP2* knockdown reduced the self-renewal ability of breast cells in both HER2- positive and triple-negative tumors. Overexpression of both *HER2* and *HER3 in vitro* increased the number of CD44^+^/CD24^–^ cells. However, knockdown of *SHP2* decreased the CD44^+^/CD24^–^ and ALDH^high^ cell population. Finally the depletion of *SHP2* in xenografts reduced tumor growth and abolished sphere formation suggesting that SHP2 contributes to the CSC phenotype (Aceto et al., 2012). Furthermore, *PTPN11* mutations have been frequently identified in glioblastomas, occurring in 7.5% of cases. SHP2 expression correlated with SOX2 expression in glioma stem cells and was decreased in the differentiated counterpart. The induction of differentiation of glioma stem cells resulted in decreased SHP2 expression (Roccograndi et al., 2017). In hepatocellular carcinoma (HCC), SHP2 promoted cell dedifferentiation and CSC expansion through the activation of β-catenin signaling (Xiang et al., 2017).

Among phosphatases, an important role in CSCs regulation has been attributed to DUSPs. Family members of DUSPs play pleiotropic and controversial roles in stemness maintenance, making their pro- or anti-cancer function particularly context-dependent. The treatment of HCC1806 BC cells with specific pharmacologic inhibitor or shRNA of DUSP9, significantly reduced DUSP9 levels and caused simultaneously reduction of the stem cells markers OCT4 and ALDH1. DUSP9 shRNA treated cells had reduced ability to form mammospheres when compared to controls. When transferred in xenograft, these cells showed reduced tumor growth compared to controls (Jimenez et al., 2020). DUSP1, DUSP4 and DUSP6 have been reported to associate with resistance to anti-cancer therapies and activation of an EMT program (Small et al., 2007; Liu et al., 2013; Boulding et al., 2016; Wu Q. N. et al., 2018). A recent study showed that DUSP6 supports a CSC phenotype in endometrial carcinoma. DUSP6 albeit inhibiting MAPK–ERK1/2 signaling, led to the activation of PI3K/Akt, with consequent increased expression of CSC-related genes in endometrial cancer cells (Kato et al., 2020). Conversely, Boulding et al. (2016) demonstrated that, although DUSP1 sustains CSC expansion, DUSP4 and DUSP6 negatively influences BCSCs maintenance. DUSP2 also showed inhibitory effects on stemness. In particular, knockdown of *DUSP2* caused an expansion of the CSC population in CRC. This cell subset showed increased expression of OCT4, NANOG, and SOX2 and tumor-sphere formation ability. In addition, DUSP2 expression levels were significantly decreased in CD133^+^ CRC cells as compared with the CD133^–^ cell counterpart (Hou et al., 2017).

## Recent Clinical Developments in Kinase and Phosphatase Inhibitors

As the knowledge obtained from different studies has shed light on the fundamental influence of kinase and phosphatase proteins in cancer biological processes, several protein tyrosine kinases and phosphatases inhibitors have granted the FDA approval or are under clinical evaluation for the treatment of various cancer types. One of the fundamental aspects for the development of molecules that inhibit protein kinases is the maximization of drug affinity for a specific target and the limited interaction with non-target enzymes. Some important physicochemical properties have to be taken into consideration for orally effective therapies. It is necessary to estimate the solubility, membrane permeability and efficacy in the drug setting. The assessment of those parameters follows the Lipinski’s “rule of five” (RO5) (Lipinski et al., 2001). Additional characteristics considered are lipophilic efficiency and ligand efficiency (Roskoski, 2021a, c).

**FIGURE 2:**
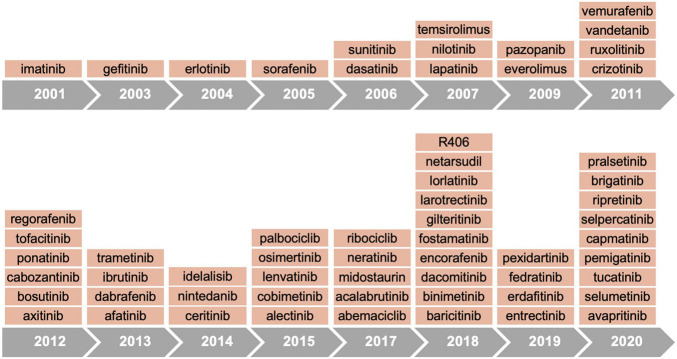
Timeline of FDA approvals for kinase inhibitors.

Since the first approval in 2001 of the imatinib, a small molecule inhibitor of ABL kinase, more than 60 protein tyrosine kinase inhibitors have received FDA authorization for cancer treatment (Figure 2 and Table 1). According to the mechanisms of action, small molecule protein kinase inhibitors have been categorized in six different classes. Type I inhibitors compete for ATP binding in the ATP-binding sites of active conformations. Type II inhibitors bind to an inactive enzyme forms and in particular to an ATP-binding adjacent site. The allosteric inhibitors, type III, interact with an allosteric site, and possess the peculiarity of being a non-competitive ATP inhibitors since they leave the ATP site free (Dar and Shokat, 2011). Type III inhibitors have been further divided into different classes: type III antagonists, which bind inside the cleft between small and large lobes close to the ATP-binding site, and type IV antagonists, which bind sites outside the ATP-binding pocket (Gavrin and Saiah, 2013). Some antagonists, classified as type V inhibitors, are defined as bivalent since they bind to separate parts of the protein kinase domain (Lamba and Ghosh, 2012). Although owing numerous advantages, including efficacy at low doses, prolonged inhibition and capability to bind targets with shallow binding sites, the scientific community has been skeptical about covalent inhibitors due to their toxicity and safety concerns. In 2013, the BTK inhibitor ibrutinib was approved for clinical use after demonstrating efficacy in lymphoma and chronic leukemia, thus leading to a new area for irreversible drugs. Subsequently, the FDA approved other six irreversible kinase inhibitors drugs (acalabrutinib, zanubrutinib, afatinib, dacomitinib, osimertinib, and neratinib) (Roskoski, 2021b).

**TABLE 1:** Protein kinase inhibitors and theirrelated targets.

**Target**						**Drug**				
ALK	Alectinib	Brigatinib	Ceritinib	Crizotinib	Gliteritinib	Lorlatinib				
BCR/ABL	Bosutinib	Imatinib	Nilotinib	Ponatinib						
BTK	Acalabrutinib	Ibrutinib								
CDK	Abemaciclib	Midostaurin	Palbociclib	Ribociclib						
C-RET	Lenvatinib	Pralsetinib	Selpercatinib							
CSF1R	Pexidartinib									
EGFR	Afatinib	Dacomitinib	Erlotinib	Gefitinib	Lapatinib	Neratinib	Osimertinib	Regorafenib	Tucatinib	Vandetanib
FGFR	Erdafitinib	Pemigatinib	Nintedanib	Lenvatinib						
FGR	Midostaurin									
FLT3	Gliteritinib	Midostaurin								
JAK	Baricitinib	Fedratinib	Ruxolitinib	Tofacitinib						
KIT	Avapritinib	Midostaurin	Pazopanib	Ripretinib						
MEK	Binimetinib	Cobimetinib	Encorafenib	Selumetinib	Trametinib					
MET	Cabozantinib	Capmatinib	Crizotinib							
m-TOR	Everolimus	Temsirolimus								
PDGFR	Avapritinib	Lenvatinib	Midostaurin	Nintedanib	Pazopanib	Ripretinib	Sorafenib			
PI3K	Idelasib									
RAF	Dabrafenib	Sorafenib	Vemurafenib							
RHO-K	Netarsudil									
ROS1	Crizotinib	Lorlatinib								
SRC	Dasatinib									
SYK	Fostamatinib	Midostaurin	R406							
TRK	Entrectinib	Larotrectinib								
VEGFR	Axitinib	Cabozantinib	Lenvatinib	Midostaurin	Nintedanib	Pazopanib	Regorafenib	Sunitinib	Vandetanib	Sorafenib

Moreover, over the past decades antibody-based therapies have significantly changed the probability of survival for oncological disorders. Some examples are further discussed below.

Besides PTK have been successfully targeted with almost a hundred of FDA-approved drugs, PTP druggability is still a major challenge due to difficulties in the design of safe and efficacious treatments (Mullard, 2018). Protein tyrosine phosphatase loss of function is due to genetic alterations including point mutations, deletion and epigenetic modification. For this reason, PTP inhibitors could comprise, in the near future, agents with different characteristics and mechanisms of action. As for now, the majority of drugs currently in clinic are mainly directed against SHP2 and PTP1B, which are central nodes for several signaling pathways. In this context, a list of phosphatase inhibitors under clinical evaluation is reported in Table 2.

**TABLE 2:** Different phosphatase inhibitors under clinical investigation.

**Drug**	**Target**	**Properties**	**Indication**	**NCT identifier**	**Status**
AKB-9778	VE-PTP	Catalytic inhibitor	Diabetic retinopathy	NCT03197870	Phase IIb
Benznidazole	PTP1B	Allosteric inhibitor	Chagas disease	NCT03378661	Phase II
GSK2983559	RIP2	Allosteric inhibitor	Inflammatory Bowel Diseases	NCT03358407	Phase I
IFB-088	PPP1R15A	Allosteric inhibitor	Charcot-Marie-Tooth	NCT03610334	Phase I
LB-100	PP2A	Catalytic inhibitor	Solid tumors	NCT01837667	Phase I
MSI-1436C	PTP1B	Non-competitive allosteric inhibitor	Breast	NCT02524951	Phase I
			Type II diabetes	NCT00606112	Phase I
PRL3-zumab	A*STAR	Monoclonal antibody	Solid tumors	NCT04118114	Phase II
RMC-4630	SHP2	Allosteric inhibitor	Solid tumors	NCT03989115	Phase I
			Leishnaniasis	NCT00662012	Phase I
SSG	SHP1, SHP2	Allosteric inhibitor	Melanoma	NCT00498979	Phase IV
			Solid tumors	NCT00629200	Phase I
TNQ155	SHP2	Allosteric inhibitor	Solid tumors	NCT04000529	Phase Ib

## Kinases and Phosphatases as Therapeutic Targets in Cancer Stem Cells

The fight against cancer has always been a challenge for the scientific community. Conventional anti-cancer therapies, as for example chemotherapy, have been pivotal treatments for cancer since the 1940s (DeVita and Chu, 2008). Over the past years, several studies have been conducted with the purpose to narrow the various chemotherapy drugs developed in order to select the best chemical compounds. Nevertheless cancer patients have received important benefits, the high mortality rate is due to chemoresistance phenomena that allow the persistence of therapy resistant cancer cell clones responsible for tumor outgrowth and metastasis formation (Turdo et al., 2019, 2021). Metastatic disease continues indeed to be a threat for cancer patients, raising the important point of the necessity of new efficacious and tailored therapies able to counteract the expansion and dissemination of the CSCs compartment. Despite the standard chemo-radiotherapy and specific kinase/phosphatase inhibitors having improved the life quality of cancer patients, metastatic disease, relapse and chemoresistance remain an outstanding issue. Hence, the discovery of kinase/phosphatase inhibitors constitute an attractive therapeutic option for the near future for the purpose to improve the efficiency of standard cancer therapy striking the refractory CSCs (Yang et al., 2020; Momeny et al., 2021).

PI3K is the major pathway considered deregulated in cancer (Fruman et al., 2017). In different types of cancer and in particular in HCC, alteration of the PI3K pathway was considered a master player for the support of CSCs. The striking increase in one of catalytic subunit type 3 of PI3K (PIK3C3) was detected in HCC tissues and liver CSCs. The inhibition of PIK3C3 hampered CSCs stability *via* the activation of AMPK. *In vitro* and *in vivo* models have shown that the combined use of PIK3C3 and PI3K inhibitor reduced spheroid formation and counteracted the tumors growth in mice models (Liu et al., 2020). In line with these observations, our group demonstrated that a subpopulation of colorectal CSCs (CR-CSC), endowed with metastatic potential, is characterized by the expression of CD44v6 and consequent activation of PI3K/Akt pathway. Targeting PI3K with the BKM120 selectively killed CD44v6^+^ CR-CSCs and reduced the growth of metastasis (Todaro et al., 2014; Veschi et al., 2020a). B591 is a novel inhibitor that targets class I PI3K isoforms, blocking the PI3K/mTOR pathway. This inhibitor proved to be effective in reducing the CSC compartment of different types of cancer. In particular, Zhou et al. (2019) observed a reduction in EMT and stemness markers, self-renewal and tumor initiating capabilities of CSCs. The combined treatment of cisplatin and the PI3K inhibitor, BEZ235, induced apoptosis of chemoresistant ovarian cancer cells. The authors also observed a reduction in colony formation capabilities, EMT and CSC markers expression (Deng et al., 2019). Akt isoforms play crucial roles in the maintenance of the CSC-like phenotype. The knock-down of the Akt1 isoform, and to a lesser extent of Akt2, affected the survival of BCSC and in particular of those displaying mesenchymal characteristics (Rivas et al., 2018). These results suggest that Akt1 is required not only for the self-renewal of CSC but also for their migratory capacity. On the other hand, glioblastoma and BCSCs deficient of Akt2 showed decreased expression of WIP and the stemness markers YAP and TAZ (Escoll et al., 2017).

Indeed, it is now clear that the PI3K/Akt pathway activation is required for the maintenance of CSCs from a variety of cancers including colorectal, ovarian, breast and hepatocellular cancer. It should not also be underestimated that this aggressive cell subpopulation could activate mechanisms of resistance to PI3K/Akt inhibitors. Of note, high HER2 expression levels have been associated with the activity of the PI3K/Akt pathway, whose targeting was not sufficient to hamper the viability of CR-CSCs. Besides targeting PI3K, with the BKM120 or taselisib, together with the HER2, inhibitor Trastuzumab and MEK inhibitors, cobimetinib or trametinib, induced the regression of tumors in CRC xenografts (Mangiapane et al., 2021).

A better understanding of the signaling pathways driven by CDKs activity is an urgent need in order to develop new specific CDKs inhibitors to block cancer cell proliferation. For instance, the role of unusual CDK, such as CDK5 was studied in glioblastoma. It was reported that CDK5 was involved in stemness of brain tumor cells in mouse models. Therefore, CDK5 enhanced asymmetric cells division and promoted glioma stem cell self-renewal through bind and phosphorylation of CREB1. The hypothesis to inhibit this kinase, with a specific inhibitor, such as CP681301, could be a change to inhibit the brain CSCs and reduce risk of recurrence (Mukherjee et al., 2018). The CDK4 positively regulates cancer stemness in triple-negative BC, where it behaved as negative prognostic marker and therapeutic target. The pharmacological inhibition of CDK4 interfered with BCSC self-renewal, promoted the transition to an epithelial phenotype and eliminated chemotherapy-resistant cancer cells (Dai et al., 2016).

An interesting example that empathizes the clinical relevance of PTP and PTK double targeting has been demonstrated by Lai et al. (2018) in the context of BCR/ABL positive leukemia patients who experienced resistance to tyrosine kinase inhibitors (TKIs). The LB100 and LB102 inhibitors of PP2A, in combination with the most effective BRC-ABL inhibitors imatinib or dasatinib (Roskoski, 2018a), reduced the viability of leukemic stem cells both *in vitro* and *in vivo*. The PP2A is implicated in human cell transformation and in cell cycle progression and its inhibition eradicated the cancer subpopulation of leukemic stem cells responsible for TKI resistance and minimal residual disease (Lai et al., 2018). These findings corroborated previous observations regarding the additive anti-cancer effect, on chronic myeloid leukemia stem cells, of the inhibition of TKs and JAK2, which is a component of the PP2A/β-catenin/BCR-ABL complex (Lin et al., 2014). However, starting from the notion that PP2A activity is inhibited by BCR-ABL, Perrotti et al. (2019) raised some concerns regarding the synergistic effect of TKIs and the PP2A inhibiting drug LB100. Several experimental flaws regarding Lai et al. (2018) study have been brought to light, including the choice to use cell lines and not primary normal and leukemia stem cells resistant to TKIs, the evaluation of cell viability rather than apoptosis and the lack of data regarding the translatability of PP2A inhibitors into clinical settings due to expected serious adverse events. Thus, the therapeutic relevance of PP2A inhibition combined with TKIs, in BCR-ABL leukemia, seems still speculative (Perrotti et al., 2019).

Triple negative BC is known as a subtype of BC more aggressive with a remarkable likelihood of relapse and metastasis. The standard treatment for this molecular subtype of BC is chemotherapy. Lu et al. (2018) have recently highlighted that the HIF-1, induced by chemotherapy, positively regulated DUSP9, while inhibiting DUSP16, in turn leading to the dephosphorylation and nuclear translocation of FoxO3 and activation of the p38 MAPK, respectively. This in turn triggers a stemness program with the enrichment of ALDH^+^ BCSCs and therapy resistance. *In vitro* treatment with the p38MAPK inhibitor SB203580 sensitized BCSCs to chemotherapy. Moreover, *in vivo* administration of another p38 MAPK inhibitor, the LY2228820, decreased tumor formation in immunocompromised mice. Interestingly, the small molecule LY2228820, has already been tested in early phases clinical trials showing tolerable side effects in advanced cancers (Patnaik et al., 2016; Lu et al., 2018).

Accordingly our group and others demonstrated that IL4, secreted by tumor and microenvironment cells interacts with IL4R expressed by cancer cells and promotes metastatic spreading by the activation of the JAK/STAT6, PI3K/Akt and MAPK pathways (Gaggianesi et al., 2017). Targeting the autocrine and paracrine IL4 signaling by using an IL4Rα antagonist (IL4DM), attenuated MAPK pathway and reduced the tumorigenic and metastatic potential of CD44^+^/CD24^–^ BCSCs. Interestingly, IL4DM treatment also potentiate the response of the immune system against the aggressive subtype of CSCs responsible for cancer progression and resistance to standard therapeutic regimens. Additionally, the inhibition of the IL4-induced NF-κB, with withaferin or 5-aminosalicylic acid (5-ASA), restored DUSP4 expression levels and consequent ERK inactivation and inhibition of stemness, invasion and proliferation of BC cells (Gaggianesi et al., 2017). Interestingly, a decrease in IL6 levels abrogated sphere-formation and *in vivo* growth of primary tumors generated by paclitaxel-resistant cervical cancer cells. In particular, the treatment with the EGFR inhibitor, erlotinib, inhibited IL6 at transcriptional and translational levels through the MUC1-EGFR-CREB/GRβ axis, causing a depletion in CSC both *in vitro* and *in vivo* (Lv et al., 2019).

The protein phosphatase WIP1 (PPM1D) inhibitors are currently under clinical evaluation for their capability to subtract p53 from proteolytic degradation or inactivation. Deng et al. (2020a) described a novel function of the WIP1 inhibitor GSK283071, which suppresses stemness features in NSCLC through the activation of p38 MAPK. Specifically, the inhibition of WIP1 induced p38 MAPK phosphorylation and consequent activation of the downstream targets MK2 and HSP27 and reduction of the SOX2, OCT4, NANOG and ALDH1A1 stemness markers, sphere forming capability and tumor-initiating potential (Deng et al., 2020a).

The small molecule BBI608 is an inhibitor of STAT-3-driven transcription, which showed cytotoxic effects against several cancer types. The peculiarity of this compounds is that it showed superiority in counteracting CSCs propagation *in vitro* as compared to other PTK inhibitors and chemotherapy. Moreover, the BBI608 depleted stem-like cells *in vivo* and prevented metastasis formation (Li et al., 2015).

The Hippo kinases Mst1/2 and Lats1/2 are responsible for the phosphorylation and inactivation of the Yes-associated protein (YAP) and the transcriptional activator with PDZ-binding domain (TAZ). The oncogenic role of YAP and TAZ has always been debated (Hong et al., 2016). Indeed, recently it has been demonstrated that YAP overactivation following the loss of LATS1/2 caused a reduction in the Lgr5^+^ CR-CSC compartment and acted as tumor suppressor role in preclinical models (Cheung et al., 2020).

The development of drugs targeting the most important oncogenic signaling, consisting in the RTKs/RAS/RAF/MEK/ERK pathway, has been the major challenge in the past 20 years (Roskoski, 2018b). One recent report aimed at demonstrating the impact of more than two thousands compounds on the activation of the Wnt pathway in colorectal cancer. While small molecules targeting BRAF and EGFR did not alter the stemness pathway, MEK1/2 inhibitors, such as trametinib, selumetinib, U0126 and PD318088, fostered an increase in Wnt signaling (Zhan et al., 2019). Accordingly, MEK inhibition induced the expression of pluripotency markers in thyroid cancer and melanoma cells (Dorris et al., 2016). On the other hand, targeting BRAF signaling pathway depleted the CD133 positive compartment in thyroid tumor (Bozorg-Ghalati et al., 2019) and synergized with cetuxiamb, an EGRF inhibitor, in reducing the CSC pool in colorectal cancer (Wu Z. et al., 2018).

## Discussion

In the present review, we propose an overview of the major kinases and phosphatases involved in many biological processes and in particular their roles in promoting tumor growth. Notwithstanding standard anti-cancer therapies have represented and actually represents the primary arm to counteract tumor bulk, the presence of a small subpopulation in the malignant cell pool, named CSCs, does not allow the total eradication of the tumor.

Therefore, CSCs are responsible for cancer relapse, higher invasiveness as well as chemoresistance.

Recent research has shown that the imbalance between kinases and phosphatases in tumor microenvironment are key elements to lead tumorigenesis, tumor growth and dissemination. In addition, gene deletions, mutations or epigenetic modifications have been shown to be crucial factors in aberrant activation of signaling pathways.

We herewith describe the latest findings regarding the contribution of kinases and phosphatases in cancer progression and dissemination. Recent advances discussed in this review regard drugs that target several kinases and phosphatases proteins deregulated in cancer. Several inhibitors have been used with the aim of counteracting advanced disease, in particular hampering CSCs.

Unfortunately, many types of advanced cancers are still appropriately untreated, in fact the challenge for the scientific community is to drive the discovery of novel therapeutic targets. Thus, there is a need to find new drugs that inhibit kinases and phosphatases proteins and, at the same time, sensitize CSCs compartments to chemotherapy.

Hereby, the aim is to enhance the efficacy of standard therapeutic approaches also using new compounds. Based on accumulated knowledge in recent years, new compounds have been used in experimental phases in patients affected by different types of cancer with promising results.

These data provide a new perspective in order to eradicate the tumor and offer a better life expectancy for the oncologic patients.

## Author Contributions

AT, CD’A, and AG contributed to design and drafting the content, figure and table in the review article. AT contributed to refine the manuscript. GPo, CC, LC, MM, NF, CM, GPi, and MB contributed to draft the manuscript. MT and GS revised the review article. All authors contributed to the article and approved the submitted version.

## Conflict of Interest

The authors declare that the research was conducted in the absence of any commercial or financial relationships that could be construed as a potential conflict of interest.

## Publisher’s Note

All claims expressed in this article are solely those of the authors and do not necessarily represent those of their affiliated organizations, or those of the publisher, the editors and the reviewers. Any product that may be evaluated in this article, or claim that may be made by its manufacturer, is not guaranteed or endorsed by the publisher.
